# A Cross-Language Study of Tonal Variants in Mandarin in Different Attentional Conditions

**DOI:** 10.3390/bs15030304

**Published:** 2025-03-04

**Authors:** Xin Chen, Jianqin Wang, Ji Lu

**Affiliations:** 1 Department of Chinese Language and Literature, Fudan University, Shanghai 200433, China; 20110110010@fudan.edu.cn; 2 Center for Cognitive Science of Language, Beijing Language and Culture University, Beijing 100083, China; 3 Key Laboratory of the Language Cognitive Science of the Ministry of Education, Beijing Language and Culture University, Beijing 100083, China

**Keywords:** second-language learners, tone variants, attentive and pre-attentive, MMN, LDN

## Abstract

This study used an electrophysiological technique to investigate the perception mechanism of Mandarin native speakers and learners from non-tonal language backgrounds when processing the third tone (T3) and its variants in Mandarin. The experiments used a 2 × 2 two-factor mixed design to examine the perception of T3 and its variants and the processing mechanisms of learners and native speakers under different levels of attention. Differences in attention and language backgrounds in the perception of Mandarin tones were further investigated. These results provide evidence that there are no significant differences in the perception of the two T3 variants by native Mandarin speakers under different attentional conditions. In contrast, learners from non-tonal language backgrounds were more likely to perceive a low flat tone as T3 than a low concave tone in the attentive condition. This means that learners are more likely to rely on low-pitch cues rather than the concave contour of the tone when perceiving T3.

## 1. Introduction

Tone refers to the variation in pitch within a syllable complemented by the different states of the vocal cords during vocalization. More than half of the world’s languages are tonal languages (Yip, 2003), and Mandarin Chinese is a typical example with four tones: a flat tone, a rising tone, a falling rising tone and a falling tone, denoted as T1, T2, T3, and T4, respectively. In the study of the evolution of languages among different ethnic groups, there is limited speculation about the reasons for the development of tones in native languages. The human brain has developed language specificity to process language during the course of evolution. For example, the left brain is more activated by time-varying sounds, such as tones and syllables, whereas the right brain is more responsive to frequency features, such as music and nonspeech sounds (Hugdahl et al., 1999). In addition, sound perception differs based on temporal relationships. Thus, there is a temporal relationship between the human brain’s speech and vocal tone processing, dividing it into pre-attention and attention phases (H. Luo et al., 2006). However, it is not clear from the current findings whether there is a switch between these two phases in activated brain regions.

In contrast to the language specificity of the brain, domain-general processing refers to the neural mechanisms underlying general cognitive tasks performed by humans. These tasks include the ability to differentiate between the pitch of two neighboring piano keys and appreciate whether a melody is melodious when processing external auditory stimuli (Giuliano et al., 2011; Fedorenko et al., 2011). However, whether speech perception involves only language specificity or both language specificity and domain generality still requires further research. It can be revealed by investigations of how second-language learners perceive their target language (TL), especially for those whose mother tongue and TL do not have much in common in terms of phonetics. If beginners perceive their TL in an acceptable way, we can deduce that domain generality plays an important role in speech perception. For Mandarin learners whose native language is non-tonal, the lack of brain specificity to process tones can lead to difficulties in tone perception and production. This often results in foreign students developing a “foreign accent” when speaking Mandarin, a topic that has received significant research attention (Lin, 1996; Lin, 2015; Zhang & Xu, 1981).

Various theories on speech perception and learning explain typical and atypical speech sounds from a categorical perspective. How do learners categorize speech sounds while learning Mandarin? What processing mechanism do learners employ in this categorization task, as reflected in brain activity through cognitive processes? In this categorization task, what type of processing mechanism, which is transmitted from cognitive processes to brain activities and then influences behavioral activities, such as articulation, is used by the learner? Essentially, confusion of tones is confusion of tonal categories. Learners from non-tonal-language do not understand the concept of tones in their native language systems, nor do they use pitch pattern changes in every syllable to differentiate word meanings. As a result, their perception and production of Mandarin tones are easily confused, and they struggle to correctly recognize and differentiate between the four Mandarin tones (Li & Zhao, 2012). Research on the perception of tonal categories has long been conducted in the field of experimental phonetics.

In recent years, studies of Mandarin tones have focused on the brain mechanisms involved in tone processing. Some studies have used electrophysiological techniques to investigate tones. The mismatch negativity (MMN) component is commonly used as an EEG index to indicate brain responses to the differences among consecutive stimuli. Most MMN experiments use the passive oddball paradigm to examine the brain’s automated processing (Näätänen, 2001; Chandrasekaran et al., 2007). Chandrasekaran found that native Mandarin speakers exhibited a higher mean amplitude of mismatched negative waves in response to Mandarin T1/T3 discrimination compared to the mean amplitude of the MMN elicited by T2/T3 discrimination. The MMN component can also be examined using the active oddball paradigm (Y. Luo & Wei, 1998; Qi, 2014). A negative wave induced by the oddball paradigm following the MMN component, found in recent years, is called the LDN (Late Discriminative Negativity) component. It responds to the participant’s further processing of the presented stimuli during the attentional process. It has the potential to be in the non-automatic and controlled processing phases, owing to the manipulation of the automatic preparatory process of the task, depending on whether there are additional changes beyond the deviant stimulus attention (H. Yang et al., 2013; Hämäläinen et al., 2008).

Each tone has a specific amount of stretch and space, although there are variations in the tonal qualities of each individual’s voice. Native speakers tend to categorize tonal variants with different vocal qualities and tonal characteristics belonging to the same tonal category, and learners do not categorize different tonal variants in the same tonal category. For example, there are many variants of Mandarin T3, with the mainstream accepted ones being rising, contour, low flat, low fall, and low concave tones. The low flat tone [11] (Shi & Ran, 2011) and the low concave tone [2112] (Cao & Wei, 2016) of T3 are currently the subject of heated debate, as illustrated in Table 1. Starting with the perception and processing mechanisms of tonal variants, this study utilized the ERP technique to systematically examine the processing mechanisms of Mandarin T3 and its variants in different attentional phases in participants with diverse language backgrounds. This was achieved by analyzing the average wave amplitudes of the MMN and LDN components. This study aimed to assist Mandarin learners in better understanding the scope of Mandarin tones and provide an experimental and theoretical basis for the advancement of Mandarin tone research and teaching.

## 2. Methods

### 2.1. Experimental Design

In this study, EEG experiments were conducted on the most controversial low flat [11] and low concave [2112] variants to investigate the automatic processing of T3 variants by learners with non-tonal language backgrounds and native Mandarin speakers in different attentional conditions. Experiments were conducted using the oddball paradigm. The first independent variable is the variant of T3, which is a within-subject variable, including low concave [2112] and low flat [11] variants. The second independent variable is the native language background, including non-tonal language background Mandarin learners and native Mandarin speakers. The third independent variable is the attentional condition, which is a within-subject variable, including attentive and pre-attentive conditions. The dependent variables are the wave amplitudes of the MMN and LDN components. To avoid learning effects, participants in the attentive and pre-attentive conditions were tested in two separate sessions, with a gap of more than three days between the two experiments for the same group of participants.

### 2.2. Participants

Fourteen native Portuguese speakers were selected (four male, ten female, mean age = 21.5, standard deviation = 2.36), along with fifteen native speakers from Beijing Language and Culture University and neighboring colleges and universities (seven male, eight female, mean age = 23.4, standard deviation = 0.81). To minimize the effects of factors that contribute to age-related hearing decline, the age range of the participants was restricted to between 18 and 28. None of the participants had hearing loss, according to their self-report. The learners were at the elementary level of Mandarin learning. None of them had passed HSK (Chinese Proficiency Test) level 4, and all of them came to China to study Mandarin for three months after studying in Portugal for a year. All of the participants were right-handed, had normal hearing, and were appropriately compensated after completing the experiment.

### 2.3. Stimuli

The syllable *yi* (/i/, “一” in Chinese characters, which means “one”) was selected as the original audio clip for the experimental material. This is because the zero-initial syllable *yi* can minimize the interference of consonants on tone and the synthesis process. The tone of the audio clip was chosen as T1 (a high-level tone), which is commonly regarded as an unmarked tone. It has a stable pitch and intensity, and its phonation type is categorized as a modal voice. Therefore, the pitch contours synthesized from this tone can represent the pitch patterns of normal phonation states, and the different tone contours have strong comparability with each other. The audio clip was recorded by a female speaker in standard Mandarin using mono-recording at a sampling rate of 44,100 Hz. The speech synthesis software Praat (Boersma, 2001, https://www.fon.hum.uva.nl/praat, accessed on 1 September 2018) was used to synthesize and modify the speech material.

**Figure 1:**
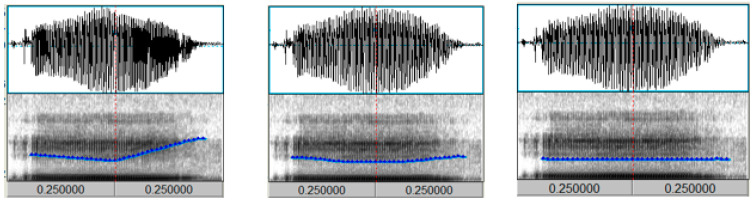
Synthesized syllable *yi* standard T3 [214] and T3 variants [2112] and [11].

Considering that the MMN components are sensitive to the frequency and duration of the stimulus material, the present study synthesized a standard T3 denoted as [214] in the five-level tone mark. Additionally, there are two variants of the T3—a low concave tone [2112] and a low flat tone [11]—with a phonetic sample of the original syllable *yi*. These variants were manipulated while keeping the intensity and length of the tone unchanged but with a change in the pitch pattern. The duration of each stimulus was normalized to 500 ms. The experimental material is shown in Figure 1.

### 2.4. Experimental Procedures

This experiment was conducted at the Key Laboratory of Language and Cognitive Science of the Ministry of Education at Beijing Language and Culture University. We ensured that the surrounding environment was quiet during the experiment. We turned off the air-conditioning exhaust and other external factors prone to noise signal interference. Before the formal experiment, the participants were required to ensure that their scalps were clean to facilitate preparation for the experiment. The experimental stimuli were presented in an auditory form using E-Prime 2.0. The participants were instructed to sit in front of a computer screen and wear in-ear air-conduit noise-canceling headphones, which also provided radiation protection. Before the experiment, the participants were briefed regarding the purpose of the experiment, which involved watching a silent movie. The participants were instructed to answer questions related to the movie at the end of the experiment. In addition, they were asked to refrain from making any body movements, blinking, or swallowing during the experiment to prevent any interference with the electrical signals essential for the experimental data. The participants were asked to sign an informed consent form prior to the experiment.

The experimental task for the pre-attentive condition involved showing the participants a silent movie and instructing them to focus on the visual content while disregarding the sound through headphones. The experimental task for the attentive condition involved asking participants to focus on the audio clips and press a button whenever they heard a sound that was not T3. The experiment was conducted using the oddball paradigm. The stimuli were categorized as standard and deviant stimuli, with presentation probabilities of 80% and 20%, respectively, where [214] was the standard stimulus and [2112] and [11] were the deviant stimuli. Stimulus intervals were randomized from 550 ms to 600 ms. The experiment initially presented 15 standard stimuli, followed by 500 standard and deviant stimuli. The experiment consisted of two experimental conditions, each with 515 trials, totaling 1030 trials. The sequence of standards and deviants was pseudo-randomized with at least two standards between two deviants.

### 2.5. EEG Recording

Experiments were conducted using a 64-conductor 10–20 system EEG device (Neuroscan Inc., Mumbai, Maharashtra, India) to record the EEG activity of participants processing Mandarin tones and their variations. The experimental procedure was recorded in real time using a Curry 8 system at a sampling rate of 1000 Hz. It involved the simultaneous recording of horizontal ocular electrograms (HEOGs) and vertical ocular electrograms (VEOGs), an online reference to the nose-tip electrode (Nz) for the recording process, an amplifier filtering bandwidth of 0.05 Hz–100 Hz, and ensuring that the impedance of all resistors was reduced to less than 5 kΩ.

### 2.6. ERP Analysis

Offline processing was performed using EEGLAB Toolbox (version 14.0.0) for baseline correction. First, the reference was set to the nose tip to enhance the efficiency of the data analysis. The sampling rate was then reduced to 500 Hz. Subsequently, the data were low-pass-filtered at 30 Hz and high-pass-filtered at 0.1 Hz. The data were segmented by intercepting them from 200 ms before stimulus emergence to 800 ms after stimulus emergence. Baseline correction was performed by setting the baseline to 200 ms before stimulus emergence. Additionally, the baseline was corrected again by manually eliminating bad segments and conductors. Independent Component Analysis (ICA) was applied to all channels to eliminate segmentation issues caused by artifacts, such as blinks and eye movements.

All data in the experiment were analyzed using the SPSS 22.0 software package. For the results of the repeated-measures ANOVA, Greenhouse–Geisser correction (Greenhouse & Geisser, 1959) was applied when the sphericity assumption was violated. The original degrees of freedom and *p*-values were reported. Multiple comparisons were tested using the Bonferroni method.

Based on previous studies, the MMN component is typically maximal at the frontal and central electrode sites (Näätänen, 1992). This study selected six electrode points, namely, F3, FZ, F4, C3, CZ, and C4, for data analysis (W. Q. Yang et al., 2020). The data were plotted and analyzed using MATLAB R2017b. The FCZ point was selected as the reference for calculating the overall average raw wave of all of the participants and generating the difference wave. Subsequently, a time window for statistical analysis was established.

## 3. Results

### 3.1. Reliability Test for MMN and LDN

#### 3.1.1. Reliability Test for Evoked MMN in Pre-Attentive Condition

**Figure 2:**
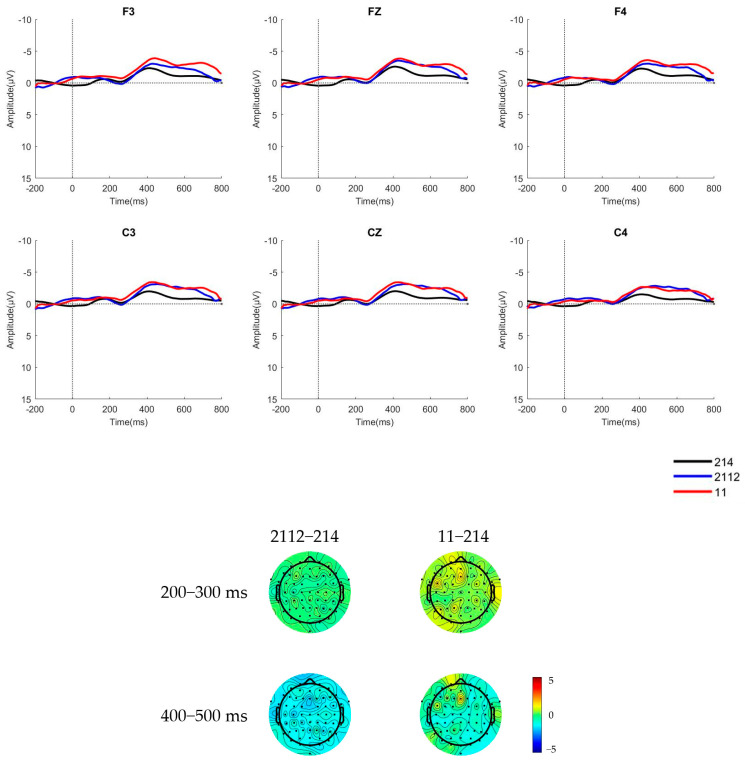
The ERP waveforms and brain topographic maps of the two stimulus types for native Mandarin speakers in pre-attentive condition.

**Figure 3:**
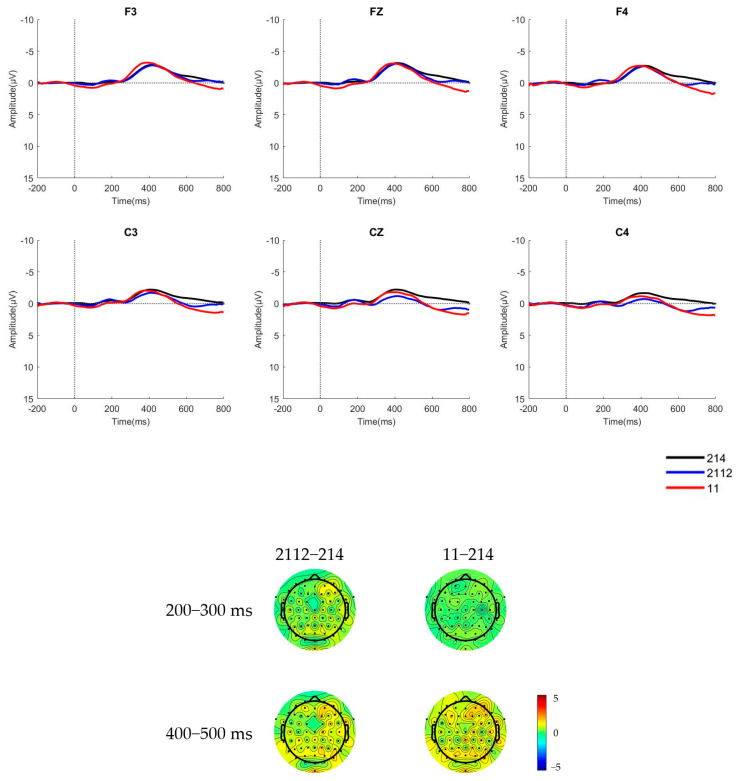
The ERP waveforms and brain topographic maps of the two stimulus types for learners of Mandarin in pre-attentive condition.

The raw waveforms of the six electrode sites and brain topographic maps of native Mandarin speakers and learners in a pre-attentive condition, where the deviant stimulus was a low concave tone [2112] and a low flat tone [11], are presented in Figure 2 and Figure 3.

The mean amplitudes and standard deviations of the difference waves of native Mandarin speakers and learners during the 200 ms–300 ms time window for MMN and 400–500 ms time window for LDN in a pre-attentive condition are shown in Table 2 and Table 3.

The cluster-based permutation test (Maris & Oostenveld, 2007) results reveal no significant differences in EEG activity across any of the analyzed time windows for Portuguese learners and native Mandarin speakers. This indicates that under the current experimental conditions, there were no statistically significant differences in EEG responses between the deviant and standard stimuli. This suggests that in the pre-attentive condition, neither group of participants evoked a significant MMN component in response to T3 and its variants.

#### 3.1.2. Reliability Test for Evoked MMN and LDN in Attentive Condition

**Figure 4:**
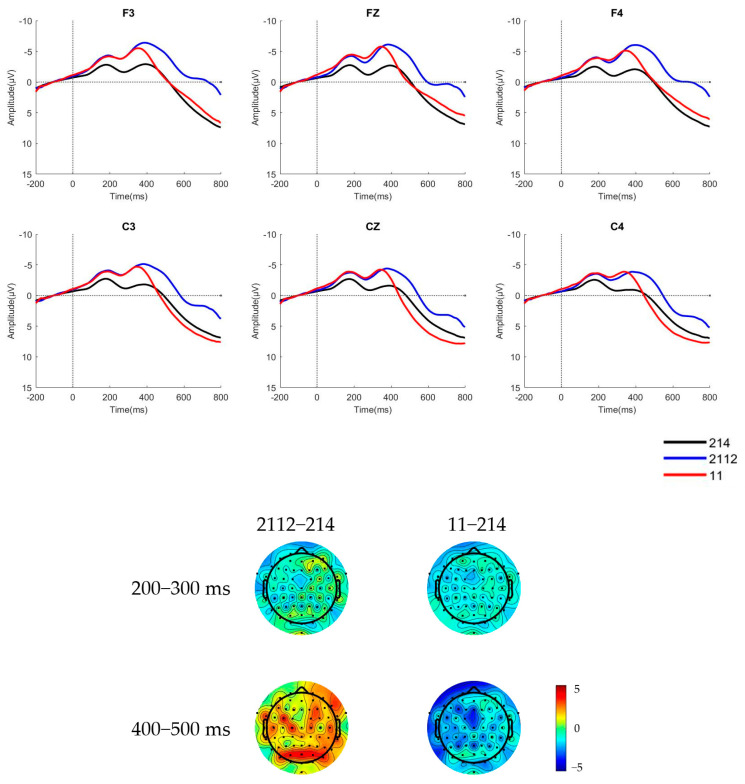
The ERP waveforms and brain topographic maps of the two stimulus types for native Mandarin speakers in attentive condition.

**Figure 5:**
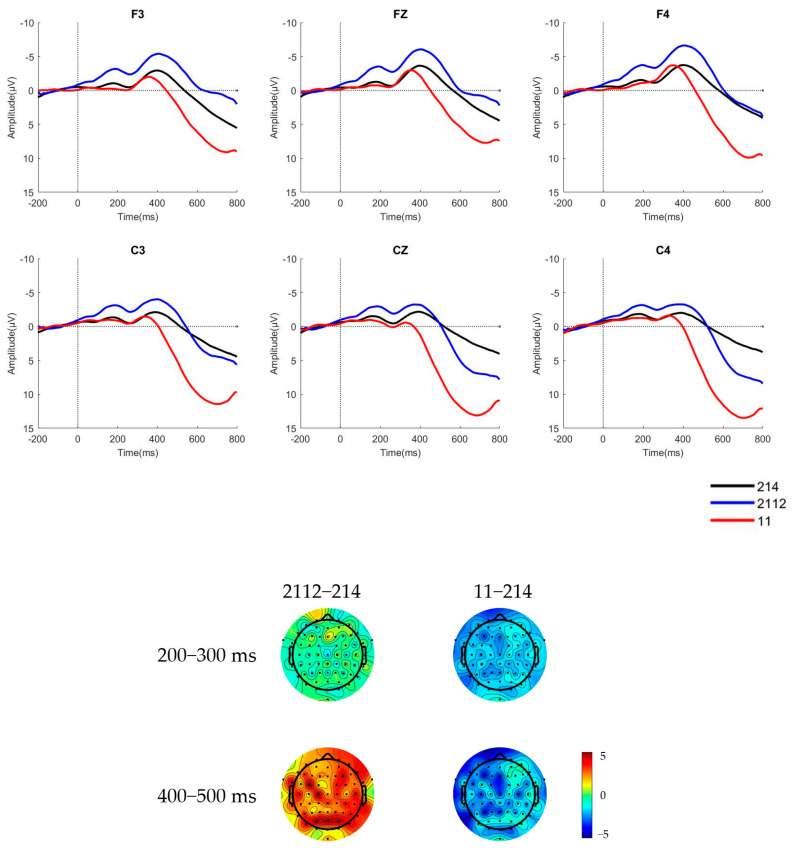
The ERP waveforms and brain topographic maps of the two stimulus types for Portuguese Mandarin learners in attentive condition.

The raw waveforms of the six electrode sites and brain topographic maps of native Mandarin speakers and learners in an attentive condition, where the deviant stimulus was a low concave tone [2112] and a low flat tone [11], are shown in Figure 4 and Figure 5, respectively.

**Figure 6:**
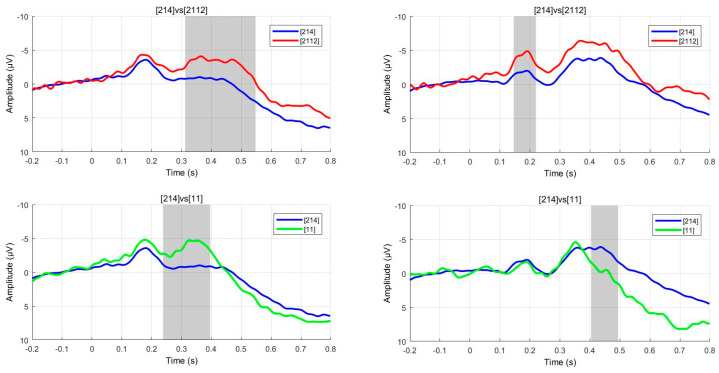
The cluster-based permutation test for native Mandarin speakers (**left**) and Portuguese Mandarin learners (**right**) in attentive condition.

The cluster-based permutation test method was employed, with 1000 permutations conducted. The cluster threshold was set at *p* = 0.05, and the significance level was α = 0.05. The permutation test results indicate significant differences in EEG activity between the two groups of participants at multiple time points and frequency bands, specifically between the low concave tone [2112] and T3 [214], as well as between the low flat tone [11] and T3 [214]. Notably, significant cluster regions were primarily concentrated within the time window of 200–500 ms. See Figure 6.

The mean amplitudes and standard deviations of the difference waves of native Mandarin speakers and learners during the 200 ms–300 ms time window for MMN and 400–500 ms for LDN in an attentive condition are shown in Table 4 and Table 5.

The analysis of variance (ANOVA) on the mean amplitude reveals that in the 200–300 ms time window, the main effect of stimulus type was non-significant, with F(1,27) = 2.461, *p* = 0.128, and η^2^ = 0.084. Similarly, the main effect of native language background was non-significant, with F(1,27) = 1.369, *p* = 0.252, and η^2^ = 0.048. However, MMN components were observed in both native Mandarin speakers and learners from non-tonal language backgrounds when perceiving the low concave [2112] stimulus. The interaction between stimulus type and native language background was marginally significant, with F(1,27) = 3.922, *p* = 0.058, and η^2^ = 0.127. The simple effects analysis indicates that Portuguese learners exhibited significantly larger difference waves in response to the low concave [2112] stimulus compared to the low flat [11] stimulus, suggesting that Portuguese learners are more likely to perceive the low flat tone as T3.

In the 400–500 ms time window, the main effect of the stimulus type remained non-significant, with F(1,27) = 2.501, *p* = 0.125, and η^2^ = 0.085. In contrast, the main effect of the native language background was significant, with F(1,27) = 5.127, *p* = 0.032, and η^2^ = 0.160. Native Mandarin speakers evoked a larger LDN component in response to the low flat [11] stimulus compared to learners. The interaction between stimulus type and native language background was non-significant, with F(1,27) = 2.202, *p* = 0.149, and η^2^ = 0.075.

### 3.2. Attentional Condition and Stimulus Type Effect Tests

An ANOVA was conducted with two attentional conditions (pre-attentive/attentive) × two stimulus types (low flat [11] stimulus/low concave [2112] stimulus) × six electrode sites (F3, FZ, F4, C3, CZ, and C4) to analyze the mean amplitude of difference waves at electrode sites within the 200–300 ms and 400–500 ms time windows. The results indicate that in the 200–300 ms time window, the main effect of stimulus type was non-significant, with F(1,28) = 0.215, *p* = 0.646, and η^2^ = 0.008. The main effect of the attentional condition was significant, with F(1,28) = 6.925, *p* = 0.014, and η^2^ = 0.198. The main effect of the electrode site was also significant, with F(5,140) = 3.975, *p* = 0.015, and η^2^ = 0.124, revealing that the frontal region evoked a larger MMN component than the central region. In the 400–500 ms time window, the main effect of stimulus type was non-significant, with F(1,28) = 0.147, *p* = 0.705, and η^2^ = 0.005. The main effect of the attentional condition was significant, with F(1,28) = 10.756, *p* = 0.003, and η^2^ = 0.278. The main effect of the electrode site was significant, with F(5,140) = 6.073, *p* = 0.002, and η^2^ = 0.178. The interaction between the electrode site and attentional condition was significant, with F(5,140) = 3.390, *p* = 0.029, and η^2^ = 0.108. In the attentive condition, the frontal region evoked a larger LDN component than the central region.

## 4. Discussion

### 4.1. Perception of the T3 Variants in Pre-Attentive Condition

Based on the experimental results, it can be seen that learners produced the MMN component when the deviant stimulus was the low concave [2112] variant. Compared to native Mandarin speakers, learners with non-tonal language backgrounds perceived the low concave [2112] variant of the Mandarin T3 with greater perceptual differences. When the deviant stimulus was the low flat [11] variant, only native Mandarin speakers exhibited an MMN component, whereas learners perceived the two tones as identical.

The MMN component observed in the experiment indicates that the ability of primary-level learners to differentiate speech sounds in the pre-attentive condition is already established when perceiving T3 and their variations in Mandarin. Moreover, the differentiation between Mandarin T3 and the low concave [2112] variant aligns with the Perceptual Assimilation Model of L2 speech learning (PAM-L2; Best & Tyler, 2007). Portuguese Mandarin learners’ native language is non-tonal. Therefore, it is challenging for them to assimilate Mandarin tonal categories into any category in their native language. However, a certain degree of similarity exists with some categories in their native language. Similar pitch contours also exist in the intonation of Portuguese, but intonation is not a phonetic category. The perceptual type corresponds to the uncategorized assimilation (UA) type in the PAM-L2 theory. With this type, both categories of the second language are easier to establish. Thus, Portuguese Mandarin learners do not differ significantly from native Mandarin speakers in their perceptions of tonal variants.

Previous studies on the perception of differences between tonal categories, such as T1/T2, T1/T3, and T2/T3, have commonly employed a passive oddball paradigm to investigate automatic processing in a participant’s brain (Morr, 2002; Chandrasekaran et al., 2007). It was found that stimulus groups with a lower degree of bias did not produce MMN components. As the present study examined the participants’ subtle perception of internal variants of tones, the two stimulus groups did not induce a strong mismatch response in the pre-attentive condition. And learners with a non-tonal language background did not evoke LDN in the 400 ms–500 ms time window. This suggests that native language background influences the neural response to tones and their variants to some extent in the late stages of attentional processing.

### 4.2. Perception of T3 Variants in Attentive Condition

Based on the experimental results, native speakers elicited the mismatch negativity (MMN) component under focused attention conditions, regardless of whether the deviant stimulus was the low concave tone [2112] or the low flat tone [11] variant. In contrast, learners from non-tonal language backgrounds only evoked the MMN component when the deviant stimulus was the low concave tone [2112] variant.

During the late stages of attentional processing, when the deviant stimulus was both native Mandarin speakers and learners from non-tonal language backgrounds, they evoked the LDN component. The mean amplitude of the wave was significantly more negative for native Mandarin speakers than for learners from non-tonal language backgrounds. Considering that low concave [2112] variants combine both modal and pitch perceptual cues, native Mandarin speakers and learners with non-tonal language backgrounds choose to process stimuli further. However, when the deviant stimuli were low flat [11] variants, neither native Mandarin speakers nor learners with non-tonal language backgrounds evoked LDN components.

Regarding the perception of T3, native speakers of Mandarin do not consider low flat [11] and low concave [2112] variants as separate tonal categories in the automated processing of the brain to distinguish them from the standard T3. Instead, they prefer to view low flat [11] and low concave [2112] variants as variants of T3. Regarding the two variants of T3, phonological experiments have shown that the “concave” nature of the low concave tone is more similar to that of T3, belonging to the same category of tones (Cao & Wei, 2016). In contrast, the low flat [11] variant lacks the typical “curved arch” shape of T3 and is classified separately from T3. The low flat [11] variant does not have the typical “curved arch” shape of T3 in terms of its tonal contour and belongs to the tones on the periphery of the T3 category (Cao & Wei, 2016). However, in the automatic processing of T3 and its variations in the brain, the two variants cannot be easily distinguished. Mandarin learners from non-tonal language countries do not categorize tones because their native languages lack such a concept. Therefore, they tend to classify the low flat [11] variant as one of T3. Consequently, learners from non-tonal language backgrounds found it easier to perceive the low flat [11] variant as standard T3.

Experiments involving participants allocating attentional resources to perceive standard T3 and T3 variants conducted with learners from non-tonal language backgrounds in an attentive condition evoked the MMN component. When confronted with subtle differences within a tonal category, such as tonal variants, the brain’s automatic processing alone does not discriminate well between the variants and the standard T3. These results support the functional hypothesis of the brain (Liberman & Whalen, 2000), indicating that early controlled processing involves attentive condition auditory signal processing, which necessitates conscious participation.

### 4.3. Suggestions for Teaching Chinese as a Foreign Language

Based on the results of the experimental study, this research offers three recommendations for teaching tones in Chinese as a foreign language.

(1) Primary-level Mandarin learners are more sensitive to the physical properties of sounds when learning Mandarin tones. The “low flat hypothesis” has become a popular teaching method, but it is challenging to detect low and flat tones in certain acoustic experiments. Therefore, researchers have proposed the low concave hypothesis for T3. In this study, we used low flat [11] and low concave [2112] variants as experimental stimuli to investigate how participants with Mandarin language backgrounds perceive these two variations in Mandarin T3. The results show that there was no significant difference in the perception of the two variants of T3 by native speakers of Mandarin in either attentive or pre-attentive conditions. In contrast, learners with non-tonal language backgrounds were more likely to perceive the low flat [11] variant as Mandarin T3 in attentive conditions. Therefore, teachers should strengthen the instruction of the tone contour in the teaching process of Mandarin T3. They should not only describe it as a low tone but also explicitly highlight its “concave” characteristic;

(2) For learners, the key to acquiring tones is to successfully establish tonal categories. Mandarin learners who are native speakers of a non-tonal language perceive T3 in pre-attentive conditions similarly to native speakers. They adopt similar processing strategies and are insensitive to the pitch changes of Mandarin tones. This confirms the UA type in the PAM-L2 theory, which suggests that learners are unable to draw an analogy between specific phonemic categories in the target language and those in their native language. In the target language, the phonemic categories were matched to those in their native language. In attentive conditions, learners from non-tonal language backgrounds exhibited different processing strategies than native speakers. There are many variants of Mandarin tones. If learners cannot establish tonal categories accurately, they may easily confuse the variants of certain tones with others. This confusion can lead to learners pronouncing tones with a “foreign accent.” Therefore, they tend to classify the low flat [11] variant as one of T3. This means that learners are more likely to rely on low-pitch cues rather than the concave contour of the tone when perceiving T3. This result is consistent with previous studies (Gao & Li, 2006; Cheng, 2006), which suggest that learners tend to rely more on pitch cues when acquiring tones;

(3) From the perspective of second-language acquisition, when a linguistic category in a second language aligns with pronunciation information already present in the native language, the learner is more capable of acquiring the phonetic category, and the native speaker perceives such foreign phonetic variants as typical phonetic content. Motor theory suggests that learners perceive speech by connecting the articulation of the language (Liberman & Mattingly, 1985). The perception of T3 in Mandarin relies on the learner’s capacity to align phonological information with muscle movements that generate speech in the brain. Consequently, learners decode foreign speech using an articulatory process. When perceiving tones, learners rely primarily on pitch cues rather than tonal cues. They distinguish tones based on the physical properties of sounds and do not categorize tones and variants in terms of linguistic properties. Therefore, Chinese language teachers should pay more attention to the linguistic properties of tones. They should not simply describe T3 as “a low tone” and teach them by displaying the values of the primary level. Rather, they need to incorporate linguistic information conveyed by pitch variations in words, phrases, and sentences to assist learners in better understanding the tonal categories. For example, incorporating gestures and visual aids or adopting specific training methods.

### 4.4. Limitations and Future Research

The findings of this study should be seen in light of some limitations. First, the sample size is smaller than similar studies due to our limited access to non-tonal language background beginners. On the other hand, it would be more credible and meaningful if we could recruit learners with tonal language backgrounds. Second, the present study only covers two variants of one single tone. Although T3 and its variants are the subject of heated debate, we should not neglect other tones, especially the second tone (T2, with a rising pitch pattern).

Variants are a universal phenomenon in spoken language to both native speakers and language learners. Our language utterances might be deviant by comparison with language norms. Such variabilities can be found in every Mandarin tone due to coarticulation (Shen, 1990) or reduction (Shih, 2021), leading to the misperception of T2 variants [44] with the standard T1 [55] for learners (Y. Wang, 1995). Some recommendations for future works are T2 and its high flat variants, T1 and its low flat variant, and whether or not the tone register carries more weight than pitch.

## Figures and Tables

**Table 1:** Pitch patterns of Mandarin T3.

Tone	Five-Level Tone Mark	Related Research
Rising tone	[14], [24], [114]	(Bradley, 1915; Liu, 1951; Gao, 2004)
Contour tone	[214], [215], [313]	(Chao, 1930; Karlgren, 1930; Lin, 1965; Shi & Wang, 2006)
Low flat tone	[11]	(L. Wang, 1979; Shi & Ran, 2011)
Low fall tone	[21], [211]	(Y. J. Wang & Li, 2010)
Low concave tone	[2112], [2113], [2114]	(Lin & Wang, 1992; Shi, 1994; Cao & Wei, 2016)

**Table 2:** Mean amplitude and standard deviation of MMN and LDN in pre-attentive condition for native Mandarin speakers.

Electrodes	MMN		LDN	
	2112–214	11–214	2112–214	11–214
	Amplitude	Amplitude	Amplitude	Amplitude
F3	0.36 (3.51)	−0.58 (3.84)	−0.08 (2.70)	−0.63 (3.67)
FZ	0.10 (3.27)	−0.49 (3.36)	−0.41 (2.42)	−0.67 (3.24)
F4	0.27 (3.37)	−0.33 (3.57)	−0.18 (2.90)	−0.54 (3.44)
C3	0.31 (2.69)	−0.48 (2.94)	−0.33 (2.05)	−0.90 (2.96)
CZ	0.16 (2.60)	−0.42 (3.11)	−0.40 (1.97)	−1.06 (3.21)
C4	0.11 (2.48)	−0.21 (3.04)	−0.51 (1.81)	−0.82 (3.02)

**Table 3:** Mean amplitude and standard deviation of MMN and LDN in pre-attentive condition for learners.

Electrodes	MMN		LDN	
	2112–214	11–214	2112–214	11–214
	Amplitude	Amplitude	Amplitude	Amplitude
F3	−0.03 (3.16)	−0.05 (2.49)	0.13 (3.79)	−0.91 (2.70)
FZ	−0.01 (3.17)	0.12 (2.56)	0.10 (3.79)	−0.51 (2.86)
F4	−0.16 (3.52)	−0.22 (2.73)	0.11 (4.12)	−0.62 (2.85)
C3	0.16 (2.58)	0.14 (2.36)	0.49 (2.87)	−0.24 (2.65)
CZ	0.40 (2.87)	0.45 (2.69)	0.95 (3.19)	−0.01 (3.02)
C4	0.39 (2.69)	0.30 (2.59)	0.95 (2.85)	0.16 (3.02)

**Table 4:** Mean amplitude and standard deviation of MMN and LDN in attentive condition for native Mandarin speakers.

Electrodes	MMN		LDN	
	2112−214	11−214	2112−214	11−214
	Amplitude	Amplitude	Amplitude	Amplitude
F3	−1.96 (2.11)	−1.99 (3.54)	−3.52 (2.93)	−3.07 (4.64)
FZ	−1.74 (2.12)	−2.43 (3.36)	−3.56 (2.88)	−3.66 (4.74)
F4	−1.87 (2.16)	−2.20 (2.99)	−3.83 (3.06)	−3.50 (4.01)
C3	−1.90 (1.81)	−1.74 (2.86)	−3.29 (2.60)	−3.14 (4.03)
CZ	−1.44 (2.16)	−1.61 (2.83)	−2.87 (2.88)	−2.96 (4.04)
C4	−1.32 (1.99)	−1.66 (2.55)	−2.74 (2.78)	−3.06 (3.86)

**Table 5:** Mean amplitude and standard deviation of MMN and LDN in attentive condition for Portuguese Mandarin learners.

Electrodes	MMN		LDN	
	2112−214	11−214	2112−214	11−214
	Amplitude	Amplitude	Amplitude	Amplitude
F3	−1.89 (3.85)	0.42 (3.43)	−2.19 (4.12)	0.33 (4.15)
FZ	−2.05 (3.87)	0.20 (2.68)	−2.34 (4.06)	−0.15 (3.49)
F4	−2.15 (4.23)	−0.21 (2.90)	−2.67 (4.26)	−0.73 (3.52)
C3	−1.66 (2.78)	0.08 (3.21)	−1.85 (3.12)	0.29 (3.58)
CZ	−1.51 (2.82)	0.53 (2.66)	−1.29 (2.73)	1.31 (3.18)
C4	−1.42 (3.01)	0.19 (2.91)	−1.35 (2.93)	0.26 (2.89)

## Data Availability

The data supporting the findings of this study are available upon request to the first author.
